# Free Women and the Birth Rate

**Published:** 1915-05

**Authors:** 


### Free Women and the Birth Rate.

Dr. Chas. V. Drysdale, of London, Secretary of the Malthusian League, has very strongly advocated woman's rights (not suffrage alone) in pointing out the relation of the birth rate to woman. He states:

"The results of present and past overpopulation may be briefly summarized as follows:

"(a) The high birth rate of thirty-five years ago simply produced a high death rate and high infantile mortality without any advantages as regards numbers. At present our birth rate is about 25, and death rate 15, and a reduction of the former to 20 would enable the latter to drop to 10 without any slackening of increase of population, but with great decrease of economic misery and infantile mortality. It may be claimed that between 20 and 30 million fewer deaths have occurred in Europe alone since 1876, as a result of the falling birth rate.

"(b) The obvious economic evils of large families have acted as a deterrent against early and general marriage, and this is the reason for prostitution.

"(c) In addition, the economic difficulties of overpopulation have led to a large amount of emigration, principally of young unmarried men. As a result of the above there has been a steadily increasing excess of women over men, which has now reached the figure of 1,360,000 in the United Kingdom (21,823,000 males, 23,183,000 females in 1909), and of which the bulk exists at the productive and marriageable ages.

"(d) The combination of the surplus of women with abstention from marriage for economic reasons has led to less than half of the women between fifteen and forty-five years of age being married, and the remainder must either remain as dependents upon their relatives or be forced into the labor market. This is the chief and fundamental reason for the entry of women into the industries, and the urgency of their demand for enfranchisement; while their competition excites sex antagonism, and their numerical excess is one of the chief reasons for male opposition to women's suffrage.

"(e) The economic pressure due to overpopulation is the chief cause of rivalry and war between nations; while the bad conditions, death, disease and physical deterioration caused by it weaken rather than strengthen nations for attack or defense, and the military spirit thus engendered is a great obstacle to the advancement of women.

"(f) The rôle of passive maternity, combined with the economic dependence of women, which is its correlate, instead of leading to the respect for women by men, which is generally pretended has had exactly the reverse effect, as it has made marriage a trap into which men have been forced to fall by sexual attraction against their intellectual judgment, and in which women have been forced to act the part of decoys. In no countries have women so much real respect as those in which their families are small, as in France, the United States, Australia and New Zealand, and, among Eastern nations, in Burma, where almost every one of our pretended safeguards for women are thrown to the winds.

"The above bold statements will, doubtless, be indignantly repudiated by a great many people, but there are many others who will realize their truth, and that not only has the principal factor in woman's subjection in the past been her passive acquiescence in unlimited maternity, but that she has thereby unwillingly inflicted a grievous harm upon the whole community. I do not personally believe that the present powerful women's movement could have arisen had it not been for the great decline in the birth rate, which has freed many women to devote themselves to this work. It is sincerely to be hoped, therefore, that feminist leaders, instead of attempting to explain away or apologize for the declining birth rate, will openly confess and glory in it, and show what a magnificent future lies before humanity when women demand their rights, as the mothers of the race to regulate their families in accordance with the possibilities of giving their children the best possible physical, mental and moral inheritance and environment, and will refuse altogether to bring weakly and diseased childred into unwholesome surroundings. When this is the case, we shall not only have improvement in every direction, racial and economic, but we shall be free from the most detestable and cowardly of all masculine hypocrisies of the present day, the claim of superiority for military service, when only 250,000 men serve in the British army and few are called on to fight, and more than a million go into the battle of maternity each year, of which 3.7 per 1000 births, or more than 4000, die on the field. No battle in the whole history of the world has had a tithe of the combatants or casualties of a single year of maternity, and there is no sadder sight than to notice the silence of women while anti-suffragists parade the abominable physical force 'argument.' This ought to show any reflecting woman how low the value of motherhood has really fallen, and convince her that the only hope of making it truly respected is to make it a limited if not a scarcity article.

"'By this we mean that the claim of Neo-Malthusianism therein developed—the voluntary regulation of the number of children by the mother—is that which secures the domestic and social individuality of the woman; that it is an incontestable and fundamental right; and that all adherents of the women's movement must reject with indignation the rule of blind chance and the old sex slavery. * * * May this book prove the incentive to this result, and may it, above all, induce our women's societies to give that serious and earnest attention to this question (hitherto so sedulously avoided) which it deserves.'

"There is no mystery about the bondwoman or the inconsistencies of the moral code to students of the population question. While marriage was only compatible with unlimited maternity, freedom was practically impossible. But science has given to women the power to break their chains, to marry the men of their choice without degrading themselves to passive annual maternity, and enveloping their loved ones in their ruin. The Bradlaugh and Besant trial of 1876 was the real signal for the advance of the freewoman, who will use and control her maternity for the glory of herself and the race."

